# PRMT1 promotes neuroblastoma cell survival through ATF5

**DOI:** 10.1038/s41389-020-0237-9

**Published:** 2020-05-15

**Authors:** Zhong-Yan Hua, Jeanne N. Hansen, Miao He, Shang-Kun Dai, Yoonjung Choi, Melody D. Fulton, Sarah M. Lloyd, Marianna Szemes, Ji Sen, Han-Fei Ding, James M. Angelastro, Xiang Fei, Hui-Ping Li, Chao-Ran Wu, Sheng-Yong Yang, Karim Malik, Xiaomin Bao, Y. George Zheng, Chang-Mei Liu, Nina F. Schor, Zhi-Jie Li, Xing-Guo Li

**Affiliations:** 1 Department of Pediatrics, University of Rochester School of Medicine and Dentistry, Rochester, NY USA; 2 Liaoning Key Laboratory of Research and Application of Animal Models for Environmental and Metabolic Diseases, Medical Research Center, Shengjing Hospital of China Medical University, Shenyang, China; 3 Laboratory of Anesthesia and Critical Care Medicine, Department of Anesthesiology, Translational Neuroscience Center, West China Hospital, Sichuan University, Chengdu, China; 4 State Key Laboratory of Stem Cell and Reproductive Biology, Institute of Zoology, Chinese Academy of Sciences, Beijing, China; 5 Department of Pharmaceutical and Biochemical Sciences, College of Pharmacy, University of Georgia, Athens, GA USA; 6 Departments of Molecular Biosciences and Dermatology, Northwestern University, Evanston, IL USA; 7 Cancer Epigenetics Laboratory, School of Cellular and Molecular Medicine, University of Bristol, Bristol, UK; 8 State Key Laboratory of Biotherapy/Collaborative Innovation Center for Biotherapy, West China Hospital, West China Medical School, Sichuan University, Chengdu, China; 9 The Georgia Cancer Center, Augusta University, Augusta, GA USA; 10 Department of Molecular Biosciences, University of California, Davis School of Veterinary Medicine, Davis, CA USA; 11 Shengjing Hospital of China Medical University, Shenyang, China; 12 Department of Pulmonary and Critical Care Medicine, Shenzhen Renmin Hospital, Shenzhen, China; 13 Department of Anesthesiology, Shenzhen Renmin Hospital, Shenzhen, China; 14 Wilmot Cancer Institute, University of Rochester School of Medicine and Dentistry, Rochester, NY USA; 15 Present Address: Department of Biology, Colgate University, Hamilton, NY USA; 16 Present Address: National Institute of Neurological Disorders & Stroke, National Institutes of Health, Bethesda, MD USA

**Keywords:** Paediatric cancer, Oncogenes

## Abstract

Aberrant expression of protein arginine methyltransferases (PRMTs) has been implicated in a number of cancers, making PRMTs potential therapeutic targets. But it remains not well understood how PRMTs impact specific oncogenic pathways. We previously identified PRMTs as important regulators of cell growth in neuroblastoma, a deadly childhood tumor of the sympathetic nervous system. Here, we demonstrate a critical role for PRMT1 in neuroblastoma cell survival. PRMT1 depletion decreased the ability of murine neuroblastoma sphere cells to grow and form spheres, and suppressed proliferation and induced apoptosis of human neuroblastoma cells. Mechanistic studies reveal the prosurvival factor, activating transcription factor 5 (ATF5) as a downstream effector of PRMT1-mediated survival signaling. Furthermore, a diamidine class of PRMT1 inhibitors exhibited anti-neuroblastoma efficacy both in vitro and in vivo. Importantly, overexpression of ATF5 rescued cell apoptosis triggered by PRMT1 inhibition genetically or pharmacologically. Taken together, our findings shed new insights into PRMT1 signaling pathway, and provide evidence for PRMT1 as an actionable therapeutic target in neuroblastoma.

## Introduction

Overexpression of protein arginine methyltransferases (PRMTs) has been well documented in various cancers and is often correlated with poor prognosis^1^. PRMT1 and PRMT5 are primary type I and II enzymes that are responsible for most asymmetrical di-methylarginine (aDMA) and symmetrical di-methylarginine (sDMA) marks, respectively^1^. Emerging evidence has linked inhibition of PRMTs to perturbation of multiple aspects of cancer cell behavior, including transformation, proliferation, invasiveness, and survival^2^. Thus, PRMTs may represent attractive cancer targets for small molecule inhibition. But it remains not well understood how PRMTs impact specific oncogenic pathways.

Our previous work demonstrated critical roles of PRMT1 and PRMT5 for neuroblastoma cell growth in part through arginine methylation of MYCN and subsequent enhancement of its stability^3,4^. Amplification of *MYCN* is found in about 25% of neuroblastoma, the most common extracranial solid tumor of childhood, and correlates with poor outcome^5^. *MYCN*-amplified tumors exhibited high levels of PRMT1 and PRMT5; knockdown of either PRMT1 or PRMT5 led to downregulation of MYCN and suppression of cell growth, suggesting a dependence of *MYCN*-amplified neuroblastoma on PRMTs^3,4^. We and others further found a significant correlation between high levels of PRMT1 and unfavorable patient outcome irrespective of *MYCN* amplification, thus implying potential MYCN-independent mechanisms for PRMT1 in neuroblastoma^3,6^.

Here, we reveal a novel role of PRMT1 in promoting neuroblastoma cell survival. We identified activating transcription factor 5 (ATF5) as a key downstream effector that mediates prosurvival function of PRMT1. We further showed that diamidine-related PRMT1 inhibitors displayed anti-neuroblastoma effects both in cell culture and in tumor-bearing mice. Our results suggest that PRMT1 may represent an attractive, druggable target for neuroblastoma.

## Results

### PRMT1 is crucial for the maintenance of murine neuroblastoma sphere cells

**Fig. 1:**
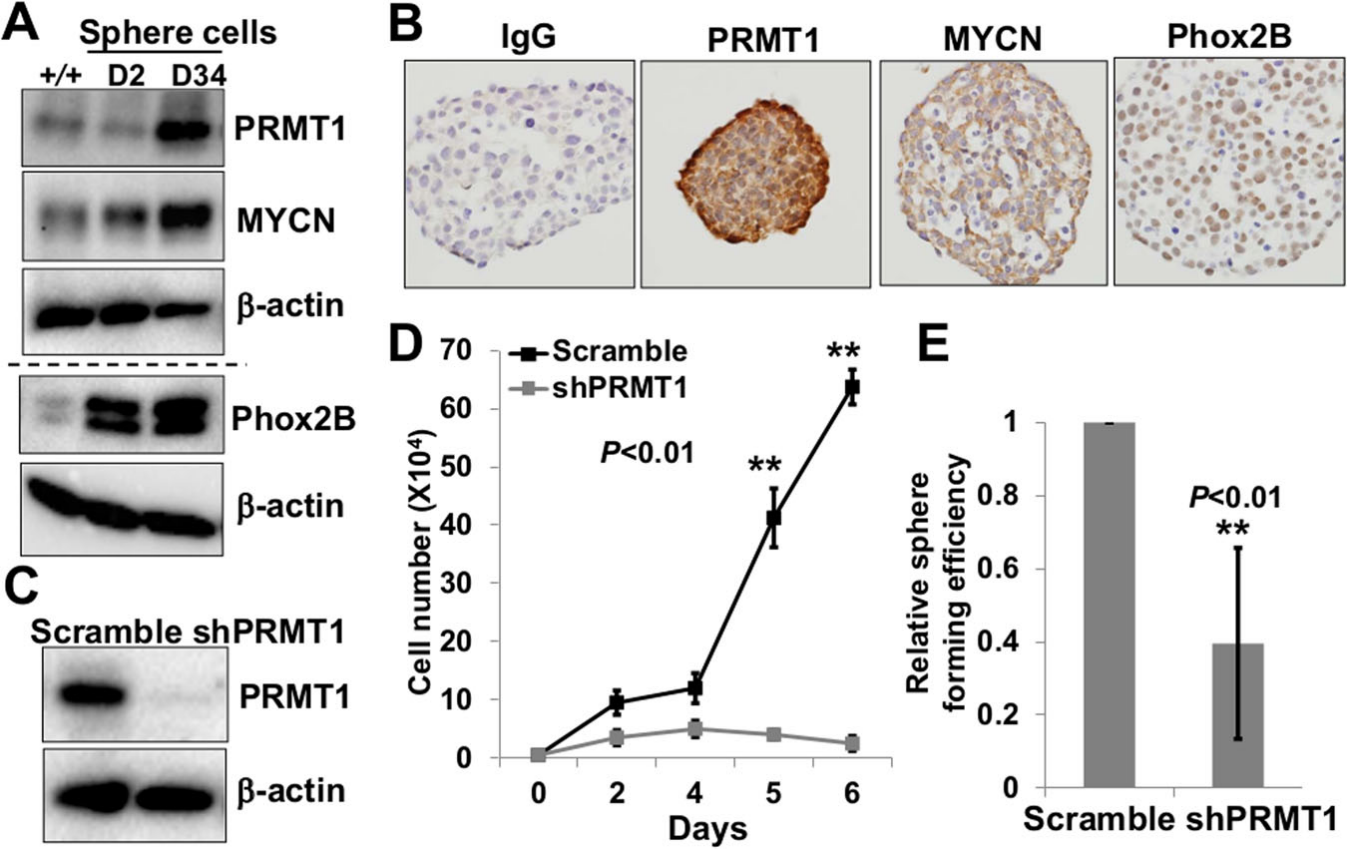
PRMT1 is required for the maintenance of murine neuroblastoma sphere cells. **a** Western blot of *Th-MYCN* primary tumors and murine neuroblastoma sphere cells (2 and 34 days in culture). **b** IHC staining in murine neuroblastoma sphere cells. **c** Western blot of murine neuroblastoma sphere cells transduced with shScramble or shPRMT1-1 lentiviruses. **d** Sphere-growth assay of murine neuroblastoma sphere cells. Data are mean ± SD (*n* = 3) relative to day 1. **e** Sphere-forming assay of murine neuroblastoma sphere cells. Data are mean ± SD (*n* = 3) relative to scramble. **P < 0.01.

Our recent studies showed that mouse neuroblastoma sphere-forming cells derived from neuroblastoma tumors in *Th-MYCN* mice possess self-renewal, differentiation, and tumorigenic potential^7^. We first confirmed that these cells exhibited self-renewal capacity both in vitro and in vivo (Supplementary Figure S1). We found that sphere cells displayed higher levels of PRMT1 and MYCN, as well as Phox2B, a specific biomarker of neuroblast progenitor cells, compared to those in primary tumors, as shown in both Western blot and immunostaining (Fig. 1a, b). Our previous observations that PRMT1 was essential for human neuroblastoma cell growth^3^ prompted us to examine whether PRMT1 is required for the growth of sphere cells. By using a previously verified shPRMT1 sequence^8^, we were able to efficiently knockdown PRMT1 in sphere cells, as shown in Western blot (Fig. 1c). PRMT1 depletion markedly inhibited sphere cell growth (Fig. 1d) and impaired their self-renewal capacity (Fig. 1e). These data suggest that PRMT1 plays an essential role in the maintenance of neuroblastoma sphere-forming cells.

### PRMT1 is essential for the proliferation and survival of human neuroblastoma cells

**Fig. 2:**
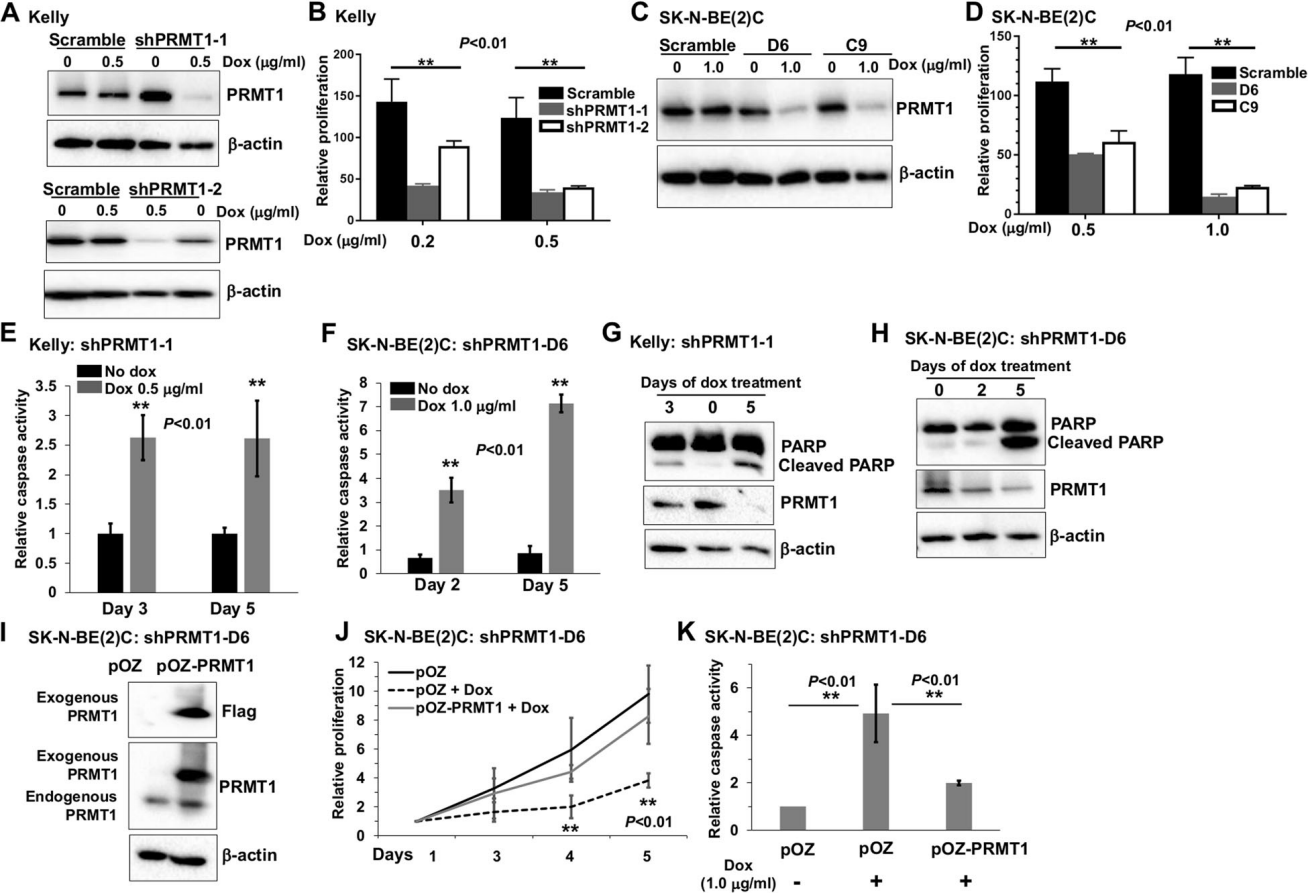
PRMT1 is essential for the proliferation and survival of human neuroblastoma cells. **a** Western blot of Kelly cells stably expressing Dox-inducible shRNA targeting PRMT1 or scramble. shPRMT1-1 and shPRMT1-2 are two independent shPRMT1 constructs. **b** PRMT1 depletion suppressed Kelly cell proliferation. Data are mean ± SD (*n* = 3) relative to scramble without Dox (100%). **c** Western blot of SK-N-BE(2)C cells stably expressing Dox-inducible shRNA targeting PRMT1-1 or scramble. D6 and C9 are two independent shPRMT1-1 clones. **d** PRMT1 depletion suppressed SK-N-BE(2)C cell proliferation. Data are mean ± SD (*n* = 3) relative to scramble without Dox (100%). Caspase-3/7 activity assay in Kelly shPRMT1-1 cells (**e**) and SK-N-BE(2)C shPRMT1-D6 cells (**f**) with or without Dox. Data are mean ± SD (*n* = 3) relative to cells without Dox. Western blot of Kelly shPRMT1-1 cells (**g**) and SK-N-BE(2)C shPRMT1-D6 cells (**h**). **i** Western blot of SK-N-BE(2)C shPRMT1-D6 cells transduced with pOZ vector or pOZ-PRMT1. **j** Cell proliferation assay of SK-N-BE(2)C shPRMT1-D6 cells transduced with pOZ vector or pOZ-PRMT1 with or without Dox. Data are mean ± SD (n = 3) relative to day 1. **k** Caspase-3/7 activity assay of SK-N-BE(2)C shPRMT1-D6 cells transduced with pOZ vector or pOZ-PRMT1 with or without Dox. Data are mean ± SD (*n* = 3) relative to pOZ without Dox. **P < 0.01.

To overcome cell growth arrest upon constitutive PRMT1 knockdown as shown in sphere cells (Fig. 1) and in human neuroblastoma cell lines described in our prior work^3^, we established inducible PRMT1 depletion by using previously validated shPRMT1 sequences^3^. Upon the addition of doxycycline (Dox), PRMT1 was efficiently depleted in *MYCN*-amplified human neuroblastoma cell line Kelly, as demonstrated by western blot in two independent shPRMT1 constructs-transduced cells (Fig. 2a). PRMT1 knockdown dramatically suppressed Kelly cell proliferation in both shPRMT1 lines (Fig. 2b), which is consistent with our previous observations in LAN-5 cells upon transient PRMT1 depletion^3^. Importantly, the critical role of PRMT1 was further confirmed in another *MYCN*-amplified human neuroblastoma cell line [SK-N-BE(2)C] using two independent shPRMT1-1 clones D6 and C9 (Fig. 2c, d). We next asked whether PRMT1 plays a similar role in *MYCN*-non-amplified cells. We then depleted PRMT1 in two independent *MYCN*-non-amplified neuroblastoma cell lines SK-N-AS and SH-EP1, and observed similar suppression of cell proliferation in both cell lines (Supplementary Figure S2A-D). Taken together, these findings indicate that PRMT1 may play a general role for neuroblastoma cell proliferation. However, we cannot rule out the possibility that MYCN may contribute to some phenotypes after PRMT1 silencing in *MYCN*-amplified cells.

To understand how PRMT1 regulates neuroblastoma cell behavior, we first evaluated effects of PRMT1 depletion on cell cycle progression. Dox-induced PRMT1 knockdown did not appear to significantly affect cell cycle progression (Supplementary Figure S2E), suggesting that the reduction of cell growth upon PRMT1 silencing might be due to an increase of cell death. To test this idea, we performed two independent experiments to investigate the effect of PRMT1 attenuation on the incidence of cell apoptosis. First, PRMT1 knockdown induced apoptosis in both Kelly and SK-N-BE(2)C cells, as evidenced by increased caspase-3/7 activity (Fig. 2e, f). Second, Western blot further confirmed the cleavage of PARP in PRMT1-depleted Kelly and SK-N-BE(2)C cells (Fig. 2g, h). These observations demonstrate that PRMT1 is a crucial factor for neuroblastoma cell survival.

To verify the specificity of shRNA-mediated knockdown, we reintroduced into SK-N-BE(2)C shPRMT1-D6 cells an empty vector (pOZ) or Flag-PRMT1 (pOZ-PRMT1) that carries silent mutations to avoid knockdown by the shRNA construct^8^. We first performed western blot of these cell lines and verified the expression of ectopic Flag-PRMT1 (Fig. 2i). As observed in the cell proliferation (Fig. 2j) and caspase-3/7 (Fig. 2k) assays, introduction of Flag-PRMT1 reversed inhibition of cell proliferation, as well as induction of caspase-3/7 activity upon PRMT1 depletion, thus excluding possible off-target effects of shRNA knockdown.

### PRMT1 promotes neuroblastoma cell survival through ATF5

**Fig. 3:**
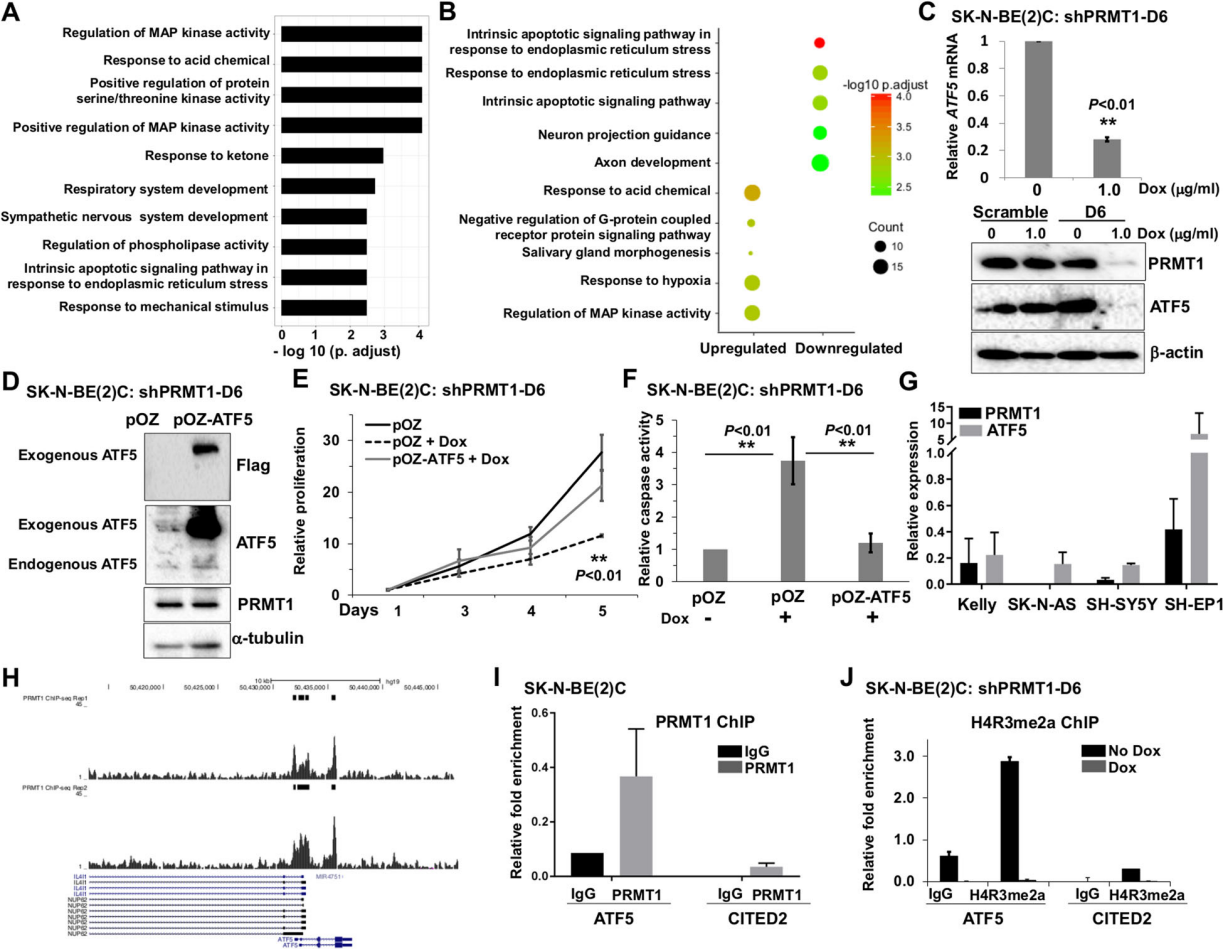
PRMT1 promotes neuroblastoma cell survival through ATF5. **a**, **b** Gene ontology analyses of differentially expressed genes in SK-N-BE(2)C cells after PRMT1 depletion. **c** qRT-PCR (top) and Western blot (bottom) of SK-N-BE(2)C shPRMT1-D6 with or without Dox. Data are mean ± SD (*n* = 3) relative to without Dox. **d** Western blot of SK-N-BE(2)C shPRMT1-D6 cells transduced with pOZ vector or pOZ-ATF5. **e** Cell proliferation assay of SK-N-BE(2)C shPRMT1-D6 cells transduced with pOZ vector or pOZ-ATF5 with or without Dox. Data are mean ± SD (*n* = 3) relative to day 1. **f** Caspase-3/7 activity assay of SK-N-BE(2)C shPRMT1-D6 cells transduced with pOZ vector or pOZ-ATF5 with or without Dox. Data are mean ± SD (*n* = 3) relative to pOZ without Dox. **g** qRT-PCR of cells transfected with ON-TARGETplus human siPRMT1 pool or ON-TARGETplus Non-targeting pool. HPRT1 was used as an internal control. Data are mean ± SD (*n* = 3) relative to cells transfected with Non-targeting pool. **h** UCSC genome browser tracks of the ATF5 gene locus showing PRMT1 binding with two different antibodies. **i** ChIP analysis of PRMT1 at ATF5 and CITED2 gene promoters in SK-N-BE(2)C cells. **j** ChIP analysis of H4R3me2a at ATF5 and CITED2 gene promoters in SK-N-BE(2)C shPRMT1-D6 cells with or without Dox. Data are mean ± SD (*n* = 3). **P < 0.01.

To define the molecular mechanisms by which PRMT1 promotes neuroblastoma cell survival, we performed RNA-seq analysis in PRMT1-depleted SK-N-BE(2)C cells to identify potential target genes and downstream signaling pathways. Gene ontology (GO) enrichment and KEGG pathway analyses of differentially expressed genes identified apoptosis and regulation of MAPK kinase activity among the most significantly regulated pathways (Fig. 3a, b). Here, we elected to focus on the prosurvival gene, activating transcription factor 5 (ATF5), one of the most highly downregulated genes in PRMT1-depleted cells. To our knowledge, there have been no previous reports on the oncogenic role of the PRMT1-ATF5 axis in neuroblastoma.

ATF5 belongs to the basic leucine zipper family of transcription factors and plays a critical role in modulating cellular differentiation and tissue development in various compartments^9^. It has been hypothesized that dysregulation of ATF5 may serve as a survival factor in cancer development, notably gliomagenesis^9^. There have been no published reports to date linking ATF5 to neuroblastoma tumorigenesis, even though the potential role of ATF5 in neuroblastoma cell survival has been implicated in recent conference abstracts. Yamashiro’s group identified ATF5 as a potential synthetic lethal gene to *MYCN*-amplified neuroblastoma^10^. Their initial studies demonstrate that ATF5 is highly expressed in *MYCN*-amplified tumors, and that silencing of ATF5 inhibits cell proliferation and induces cell apoptosis^10,11^. They further show that an ATF5-targeting peptide has profound in vitro and in vivo cytotoxic and apoptotic effects on neuroblastoma^11^.

We report here, to our knowledge, PRMT1 as a novel regulator of ATF5 in neuroblastoma. We first performed RT-qPCR analysis to verify the downregulation of ATF5 mRNA expression in PRMT1-depleted cells (Fig. 3c). Notably, PRMT1 ablation reduced ATF5 expression at the protein level as well (Fig. 3c), confirming that PRMT1 is required for ATF5 expression and might be an upstream activator of ATF5 expression. Furthermore, we also observed a marked decrease of ATF5 mRNA in another *MYCN*-amplified cell line Kelly after PRMT1 silencing (Fig. 3g). To test the functional role of ATF5, we overexpressed Flag-tagged ATF5 in SK-N-BE(2)C shPRMT1-D6 cells and verified the expression of ectopic ATF5 (Fig. 3d). Next, we performed two independent assays to examine the functional requirement of ATF5 in PRMT1-mediated survival pathway. First, we monitored cell proliferation by using Alamar blue assay and found that ectopic expression of ATF5 rescued the inhibition of cell proliferation upon PRMT1 silencing (Fig. 3e). Second, we evaluated cell apoptosis by using caspase-3/7 activity assay and observed that Dox-mediated PRMT knockdown induced cell apoptosis in pOZ vector-transduced cells, but not in cells ectopically expressing ATF5 (Fig. 3f). These results indicate that ATF5 is a downstream functional effector of PRMT1-mediated survival pathway.

Given that our prior study implicated MYCN as an important target of PRMT1^3^, we are aware of the potential contribution of MYCN to the gene expression changes after PRMT1 silencing in *MYCN*-amplified cells. In addition, ATF5 was recently identified as a potential synthetic lethal gene to MYCN^10^, raising the possibility that ATF5 may be directly or indirectly regulated by MYCN. To test whether PRMT1 regulates ATF5 expression independent of MYCN, we measured ATF5 expression in three *MYCN*-non-amplified cell lines after PRMT1 silencing, two of which, SK-N-AS and SH-SY5Y displayed a marked decrease of ATF5 expression upon PRMT1 depletion (Fig. 3g). Importantly, PRMT1 silencing also suppressed cell proliferation in these cell lines (Supplementary Figure S2A-D). It is possible that MYCN may contribute to the regulation of ATF5 in *MYCN*-amplified cells. Nevertheless, these results support the notion that the PRMT1-ATF5 axis plays a critical role in neuroblastoma cell growth and survival, regardless of *MYCN* amplification status.

We next set out to evaluate the mechanisms by which PRMT1 regulates *ATF5* expression. We have previously demonstrated a cross-talk between H4R3me2a mark deposited by PRMT1 and subsequent histone acetylation, as well as the recruitment of general transcription machinery^8,12^. These findings lead us to hypothesize that PRMT1 may activate ATF5 transcription through modulating H4R3me2a mark. First, to assess whether PRMT1 binds to the ATF5 locus, we retrieved our recent ChIP-seq results in human keratinocytes expressing HA-PRMT1^13^. By using two different antibodies, we observed PRMT1 peaks that were enriched at the ATF5 gene locus (Fig. 3h). Importantly, ChIP-qPCR demonstrated enrichment of PRMT1 at *ATF5* gene promoter in SK-N-BE(2)C cells, but not at *CITED2* gene promoter whose mRNA level did not change in PRMT1-depleted cells (Fig. 3i). Finally, ChIP further demonstrated that silencing of PRMT1 dramatically reduced H4R3me2a enrichment at *ATF5* gene promoter, but not at *CITED2* gene promoter where H4R3me2a was not enriched (Fig. 3j). Taken together, these data indicate that PRMT1 promotes cell survival through modulating H4R3me2a mark at *ATF5* gene and thus activating its transcription and prosurvival activity. It is important to note that additional experiments are needed to test whether PRMT1 directly regulates ATF5 transcription. For instance, the unspliced form of ATF5 mRNA should be measured upon PRMT1 silencing. Furthermore, a luciferase reporter mini-gene containing or not containing ATF5 promoter regions bound by PRMT1 should be used in stably transfected *MYCN*-non-amplified cells to examine the reporter activity and H4R3me2a enrichment in the presence or absence of PRMT1.

### Diamidine compounds inhibit neuroblastoma cell growth

**Fig. 4:**
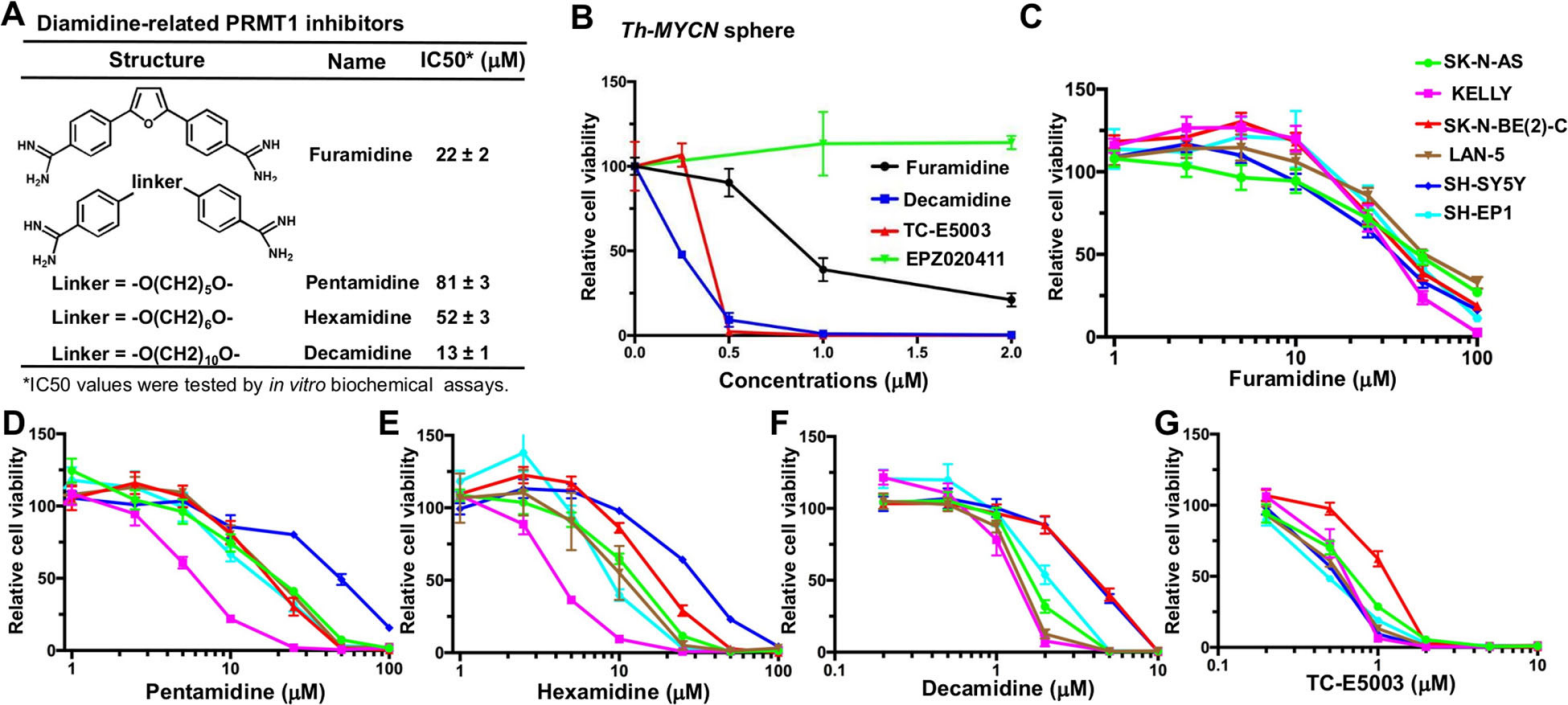
Diamidine compounds reduce cell growth in murine neuroblastoma sphere cells and human neuroblastoma cell lines. **a** A list of diamidine-related PRMT1 inhibitors. IC50 values were previously determined by in vitro biochemical assays^16^. **b** Cell viability of murine neuroblastoma sphere cells treated with indicated compounds for 3 days. Cell viability was determined by using Alamar blue assay and normalized to DMSO-treated cells (100%). **c**–**g** Cell viability of human neuroblastoma cells treated with indicated compounds for 2 days (**c**, furamidine; **d**, pentamidine; **e**, hexamidine; **f**, decamidine; **g**, TC-E5003), as determined by using Alamar blue assay. Data are mean ± SD (*n* ≥ 3) relative to DMSO control (100%).

To date, pharmacological inhibition of PRMT1 activity in tumors has not been fully explored largely due to limited specificity and selectivity of most currently available inhibitors^14^. Focused library screenings by us and others have identified low micromolar PRMT1 inhibitors, including the diamidine compound furamidine and its analogs^15–18^ (Fig. 4a). We have studied the structure-activity relationships of diamidine-related compounds by assaying their ability to inhibit neuroblastoma cell growth^19^. First, we found that treatment with two of diamidine compounds, furamidine and decamidine led to a concentration-dependent reduction of cell viability in murine neuroblastoma sphere cells with decamdine exhibiting much higher potency than furamidine (Fig. 4b). TC-E5003, a selective PRMT1 inhibitor that does not belong to the diamidine family^20^ also displayed suppression of sphere cell viability similar to decamidine (Fig. 4b), thus supporting that PRMT1 activity plays an essential role in neuroblastoma sphere cells. Importantly, EPZ020411, a PRMT6-selective inhibitor^21^, did not affect sphere cell viability at the highest concentration tested (Fig. 4b). Next, we expanded our studies to a wide panel of human neuroblastoma cell lines, including *MYCN*-amplified [SKN-BE(2)-C, LAN-5, and KELLY] and *MYCN*-non-amplified (SH-SY5Y, SK-N-AS, and SH-EP1) cell lines (Fig. 3c–g). The cytotoxicity of diamidine compounds largely correlated with their in vitro PRMT1 inhibitory effects as determined by biochemical assays with synthetic PRMT1 substrates^15^. The inhibitory effect on neuroblastoma cell viability follows the rank order decamidine > TCE5003 > hexamidine > pentamidine > furamidine. Interestingly, these compounds exhibited similar potency in *MYCN*-amplified versus non-amplified neuroblastoma cell lines, which is consistent with a similar reduction of cell proliferation upon PRMT1 silencing regardless of *MYCN* amplification (Fig. 2 and Supplementary Figure S2). In addition, we noticed that many, if not all, diamidine compounds showed much higher potency in sphere cells than in neuroblastoma cell lines. This differential potency could be simply due to different species between mouse neuroblastoma-derived sphere cells and human neuroblastoma cell lines. Another possible scenario is that a small cancer stem-like subpopulation in sphere cells may present unique vulnerability to PRMT1 inhibition. To test this idea, further studies are needed to compare the potency of PRMT1 inhibitors in isolated neuroblastoma cancer stem cell fraction based on certain surface markers (i.e., CD133 and FZD6) to that in neuroblastoma cell lines from the same species. Whether and how PRMT1 plays differential roles for survival of neuroblastoma spheres versus cell lines awaits further investigation. Nevertheless, our results are consistent with the notion that, despite several known activities of diamidine-related compounds^14^, their anti-proliferative activity in neuroblastoma cells relates, at least in part, to their inhibition against PRMT1.

### Pharmacological inhibition of PRMT1 induces neuroblastoma cell apoptosis

**Fig. 5:**
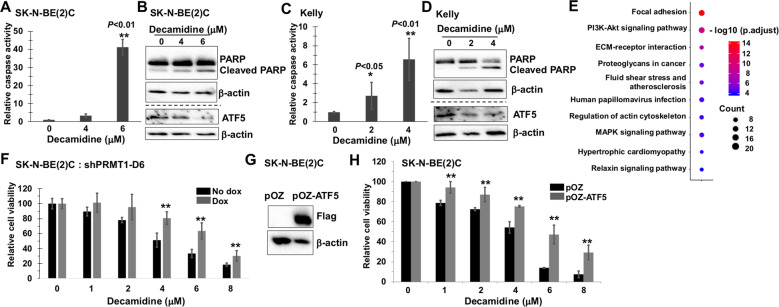
Pharmacological inhibition of PRMT1 suppresses cell growth and induces cell apoptosis in human neuroblastoma cells. **a** Caspase-3/7 activity assay of SK-N-BE(2)C cells treated with indicated concentrations of decamidine for 24 h. Data are mean ± SD (*n* = 3) relative to DMSO control. **b** Western blot of cells in (**a**). **c** Caspase-3/7 activity assay of Kelly cells treated with indicated concentrations of decamidine for 24 h. Data are mean ± SD (*n* = 3) relative to DMSO control. **d** Western blot of cells in (**c**). **e** Gene ontology analysis of differentially expressed genes in SK-N-BE(2)C cells following 24 h of treatment with 4-μM decamidine. **f** PRMT1 knockdown altered the sensitivity of SK-N-BE(2)C cells to decamidine. SK-N-BE(2)C shPRMT1-D6 cells were treated with or without Dox for 3 days, followed by 24 h of treatment with indicated concentrations of decamidine. Cell viability was determined by using Alamar blue assay. Data are mean ± SD (*n* = 3) relative to DMSO control (100%). **g** Western blot of SK-N-BE(2)C cells transduced with pOZ vector or pOZ-ATF5. **h** Cell viability of SK-N-BE(2)C cells stably expressing pOZ vector or pOZ-ATF5 treated with indicated concentrations of decamidine for 24 h. Data are mean ± SD (*n* = 3) relative to DMSO control (100%). *P < 0.05; **P < 0.01.

To determine molecular mechanisms of cell growth inhibition by diamidine compounds, we chose the most potent diamidine compound, decamidine and first asked whether decamidine induces apoptosis in cell culture. Indeed, in two independent human neuroblastoma cell lines SK-N-BE(2)C and Kelly, we observed an increase of caspase-3/7 activity, as well as cleaved PARP upon treatment with low micromolar concentrations of decamidine (Fig. 5a–d). Notably, western blot indicated a dose-dependent decrease of ATF5 protein level in both cell lines (Fig. 5b, d). These data suggest that pharmacological inhibition of PRMT1 activity phenocopies genetic ablation of PRMT1 toward downregulation of prosurvival signaling and induction of cell apoptosis. To gain direct insight into cellular mechanisms of decamidine, we treated SK-N-BE(2)C cells with 4 μM decamidine for 24 h and examined transcriptional changes using RNA-seq. Among the top 10 significantly regulated pathways, PI3K-Akt signaling pathway, ECM-receptor interaction, and MAPK signaling pathways were the most differentially expressed (Fig. 5e), suggesting that decamidine treatment may partially recapitulate effects of PRMT1-specific genetic ablation, as well as trigger other downstream effects that are PRMT1-independent (Fig. 3a, b).

To further investigate whether the anti-proliferative effect of diamidine compounds is due to PRMT1 inhibition, we examined the sensitivity of cells to decamidine under PRMT1 knockdown conditions. If decamidine inhibits cell growth by solely targeting PRMT1, knockdown of PRMT1 would increase the sensitivity of cells to decamidine. Intriguingly, we found that PRMT1 silencing dampened the cytotoxicity of decamidine in SK-N-BE(2)C-shPRMT1-D6 cells (Fig. 5f). A likely explanation is substrate scavenging previously observed upon loss of PRMT1^22^. In *Prmt1*-null cells, there are major increases in global sDMA marks that are mainly deposited by PRMT5^22^. In good agreement with previous studies, we observed in multiple and diverse PRMT1-depleted human neuroblastoma cell lines an increase of sDMA levels along with the expected decrease of aDMA levels, with no detectable change of PRMT5 protein level (Supplementary Figure S3A-E). This observation is consistent with the notion that loss of PRMT1 activity impacts substrate protein function by switching to a different methylation type mediated by PRMT5^22,23^. Indeed, recent studies have demonstrated a redundancy between PRMT1 and PRMT5 pathways and synergistic anti-tumor effects of combined inhibition of both PRMTs^23–25^. In the context of neuroblastoma, knockdown of only PRMT1 or PRMT5 leads to cell apoptosis (Fig. 2 and ref. ^4^). In addition, decamidine was shown to bind to PRMT1 and PRMT5 similarly^15^. Taken together, it is tempting to speculate that the increased level of sDMA in PRMT1-depleted cells would require more decamidine for inhibition of PRMT5 activity. However, we cannot rule out the possibility that other molecular substrates, in addition to PRMT5, the activity of which is modulated upon PRMT1 silencing, might induce a prosurvival effect and thus counterbalance the loss of PRMT1.

Finally, we investigated whether ATF5 mediates the anti-proliferative effect of diamidine compounds. We generated stable SK-N-BE(2)C cell lines overexpressing pOZ vector or pOZ-ATF5 and validated the expression of ectopic ATF5 (Fig. 5g). We observed a significant resistance of ATF5-overexprssing cells to decamidine compared to the vector-expressing cells at all the concentrations tested (Fig. 5h). Therefore, the expression level of ATF5 is associated with sensitivity to diamidine compounds and ATF5 functions as a downstream effector of PRMT1 signaling to promote cell survival. Collectively, these results provide multiple lines of evidence supporting the specificity of diamidine compounds toward suppression of the PRMT1-ATF5 survival signaling axis in neuroblastoma.

### PRMT1 inhibitors impair the growth of established neuroblastoma tumors

**Fig. 6:**
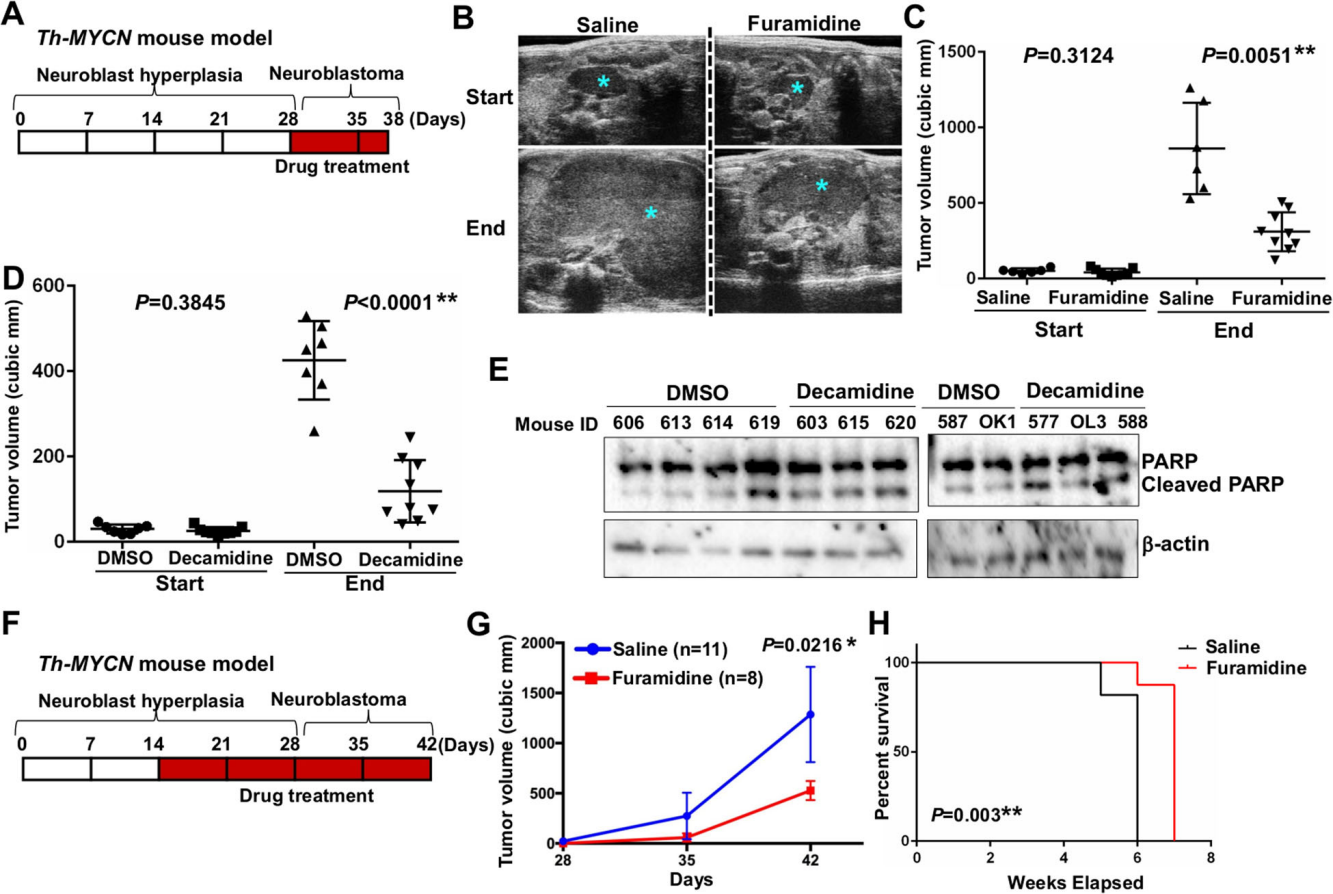
Diamidine compounds impair neuroblastoma tumor growth in vivo. **a** Schematic representation of progression of spontaneously developing neuroblastoma tumor in homozygous *Th-MYCN* mice. The numbers shown are postnatal days. The drug treatment started from P28 for 10 days. **b** Representative ultrasound images of mice treated with saline or furamidine at P28 (start) and P38 (end). Tumors are indicated by asterisks. **c** Tumor volume of mice treated with saline (*n* = 6) or furamidine (*n* = 9) at start and end points as determined by ultrasound imaging. **d** Tumor volume of mice treated with DMSO (*n* = 7) or decamidine (*n* = 9) at start and end points as determined by ultrasound imaging. **e** Western blot of tumor lysates from (**d**) at the end point. **f** The drug treatment started from P14 for 4 weeks and the tumor growth was assessed weekly by ultrasound imaging. **g** Tumor volume of mice treated with saline (*n* = 11) or furamidine (*n* = 8). **h** A Kaplan–Meier plot for survival of mice in (**g**) treated with saline or furamidine considering time to tumor burden (500 cubic mm by ultrasound imaging). Log-rank test was used to calculate statistical significance between saline- and furamidine-treated tumors.

The *Th-MYCN* transgenic mice carry in the germline human MYCN driven by a promoter of the rat tyrosine hydroxylase gene (*Th*) which is expressed specifically in neural crest lineage cells^26^. These animals spontaneously develop neuroblastoma tumors that recapitulate many of histological and pathological aspects of human neuroblastoma^26^. Four-week-old tumor-bearing *Th-MYCN*^*+/+*^ mice were treated with saline or furamidine (10 mg/kg) daily for 10 days and the tumor volume was monitored by ultrasound imaging (Fig. 6a, b). This dose of furamidine did not cause a significant weight loss (Supplementary Figure S4A-B). Treatment with furamidine significantly suppressed tumor growth compared to the saline control (Fig. 6c). Next, we extended these studies to two more potent diamidine compounds, hexamidine and decamidine. The doses we used (10 mg/kg of decamidine) were well tolerated without causing a significant weight loss (Supplementary Figure S4C-D). Consistent with the higher in vitro potency of hexamidine and decamidine as compared to furamidine, administration of hexamidine or decamidine dramatically diminished tumor growth of established neuroblastoma tumors (Fig. 6d, Supplementary Figure S4E-F). Importantly, assessment of tumor lysates from mice treated with decamidine demonstrated tumor cell apoptosis, as evidenced by elevated amounts of cleaved PARP (Fig. 6e). Furthermore, we employed an independent preclinical xenograft model of SK-N-BE(2)C cells and decamidine treatment resulted in decreased tumor growth (Supplementary Figure S4G). Taken together, these results suggest that PRMT1 inhibition with diamidine compounds may be an effective therapeutic strategy for neuroblastoma.

### Perinatal inhibition of PRMT1 activity suppresses neuroblastoma tumor initiation and progression

Finally, we examined whether suppression of PRMT1 activity could block neuroblastoma tumor initiation. We initially treated seven-day old *Th-MYCN*^*+/+*^ mice with furamidine at 10 mg/kg for 4 cycles of 5 days on/2 days off and observed improved animal survival, as well as a severe loss of body weight (data not shown). We then administered furamidine at 10 mg/kg from 2 weeks of age for 4 cycles (Fig. 6f). Mice treated with this regimen did not show a significant weight loss and no other obvious toxicity was observed (Supplementary Figure S4H-I). Treatment with furamidine before tumor initiation significantly inhibited subsequent tumor growth, reduced tumor volume at the end of treatment by ~50% (Fig. 6g), and extended animal survival (Fig. 6h). Together, these data confirm a critical role of PRMT1 activity for the initiation and progression of neuroblastoma tumor in a well-established preclinical mouse model and suggest that inhibition of PRMT1 activity may represent a promising strategy for neuroblastoma.

## Discussion

Here we present evidence supporting an essential function of PRMT1-mediated survival pathway in neuroblastoma. Given multiple roles of arginine methylation, the functional complexity of PRMTs is mainly reflected by a large number of histone and non-histone substrates involved in diverse cellular processes. Indeed, our prior studies identified arginine methylation of MYCN and subsequent enhancement of MYCN stability by PRMT1 and PRMT5 in neuroblastoma^3,4^. Our current results reveal a new role for PRMT1 to promote cell survival through modulating H4R3me2a mark at the prosurvival gene *ATF5*. Notably, neuroblastoma cells are sensitive to PRMT1 inhibition irrespective of *MYCN* amplification, suggesting that additional biomarkers of response to suppression of PRMT1 activity are likely to be relevant. We propose that PRMT1 may exert its functions through multiple and non-mutually exclusive mechanisms to stimulate neuroblastoma cell growth.

In neuroblastomas with *MYCN* amplification or high MYCN levels without *MYCN* amplification, the positive regulatory loop of the MYCN-PRMT1 axis may promote a MYCN-driven oncogenic program^3,6^ whereas PRMT1-mediated activation of ATF5 may lead to enhanced survival. We speculate that there may be cross regulatory mechanisms between PRMT1-MYCN and PRMT1-ATF5 axes. This idea is supported by initial findings by Yamashiro’s group demonstrating ATF5 as a potential synthetic lethal gene to *MYCN*-amplified neuroblastoma^10^, although the mechanistic link between MYCN and ATF5 remains to be characterized. On the other hand, a similar role of the PRMT1-ATF5 axis may apply to neuroblastomas with low MYCN levels. This notion is supported by our current work revealing downregulation of ATF5 in PRMT1 knockdown cells regardless of *MYCN* amplification status, and similar sensitivity in *MYCN-*non-amplified neuroblastoma cells to PRMT1 inhibition as that in *MYCN*-amplified cells, as well as by initial studies of ATF5 in neuroblastoma^11^. Yamashiro’s group found that suppression of ATF5 activity with an inhibitory peptide led to a concentration-dependent decrease of cell viability and increase of cell apoptosis in a panel of human neuroblastoma cell lines regardless of *MYCN* amplification^11^. Therefore, it is tempting to speculate that ATF5 may act as a general downstream effector of PRMT1-mediated survival signaling in neuroblastoma. Active investigations are now underway to test this hypothesis. However, we cannot rule out the possibility that MYCN may directly or indirectly regulate ATF5 in *MYCN*-amplified cells. Furthermore, it is noteworthy that PRMT1 has been recently shown to modulate cellular senescence and migration activity in a non-*MYCN*-amplified neuroblastoma SK-N-SH cell line^27^. Seminal studies have demonstrated phenotypically divergent subclones that are derived from SK-N-SH cell line and that these subclones may interconvert^28,29^. Our results indicate a prosurvival role of PRMT1 in a panel of human neuroblastoma cell lines comprising of major phenotypically divergent cell types. Further dissection of downstream signaling events of PRMT1 is needed to fully reveal the multifaceted complexity of this epigenetic factor as a regulator of neuroblastoma.

Until recently, small molecule inhibition of PRMT1 has not yet been fully explored in cancer therapy largely due to issues pertaining to specificity, selectivity, potency, and cellular permeability^1,14^. In our current study, multiple lines of evidence have verified these properties of diamidine compounds. However, as with many targeted compounds, targeting epigenetic modifiers as a monotherapy often results in only modest therapeutic effects. We have noted from our in vivo studies that PRMT1 inhibition alone may not be sufficient to completely ablate tumor formation and progression. Interestingly, recent studies have provided a rational combinatorial inhibition strategy of type I PRMTs and PRMT5 for synergistic killing of cancer cells^23–25^. The dual inhibition of PRMT1 and PRMT5 has not yet been evaluated in neuroblastoma. We predict that simultaneous targeting of PRMT1 and PRMT5 may prove more efficacious based on the following reasons. Frist, PRMT1 and PRMT5 may share common substrates that are similarly regulated by both aDMA and sDMA marks. Our prior identification of MYCN as a shared substrate for PRMT1 and PRMT5 suggests that arginine methylation of MYCN plays a critical role for the stabilization of MYCN and subsequent MYCN-driven oncogenic program^3,4^. The mechanistic interplay between aDMA and sDMA of MYCN is currently unclear, but it appears that a certain threshold of arginine methylation regardless of whether it is aDMA or sDMA is required for cell survival, as demonstrated by induction of cell apoptosis in cells depleted of either PRMT1 or PRMT5 through RNA interference^3,4^. This idea is supported by a recent report showing that MYC is both asymmetrically and symmetrically dimethylated by PRMT1 and PRMT5, respectively, with different functional properties in glioblastoma^30^. Second, the high potency of decamidine we observed in our current study may be due to combined PRMT1 and PRMT5 inhibition. Compared with all the other diamidine compounds, decamidine possesses the most potent inhibitory effect toward PRMT1, but slightly decreased selectivity against PRMT5^15^. It is likely that simultaneous inhibition of PRMT1 and PRMT5 by decamidine on common or distinct substrates, such as MYCN or MYC brings the overall arginine methylation levels below the threshold required for cell growth and survival. Intriguingly, we observed an increased resistance to decamidine in PRMT1 knockdown cells. This is likely due to substrate scavenging by other PRMTs upon loss of PRMT1^22^ and the compensatory accumulation of sDMA and MMA marks may render PRMT1-depleted cells more resistant to decamidine. It is important to note that the synergy between PRMT1 and PRMT5 results from a complicated cross-talk between PRMT1 and PRMT5 on many under-characterized substrates and through diverse downstream signaling events^23–25^. Indeed, we found that the currently available PRMT5 inhibitors, such as EPZ015666 were not able to recapitulate the sensitivity of human neuroblastoma cells in response to depletion of PRMT1 or PRMT5 (data not shown). This discrepancy between genetic depletion and pharmacological inhibition of PRMTs has been previously described in a number of cancers and warrants further investigation^23–25^. Importantly, our studies indicate that regulation of MAPK kinase activity, separate from apoptosis pathway, is among the most significantly regulated effects of PRMT1 depletion on neuroblastoma cells. This is critically important, as neuroblastomas at relapse harbor an increased spectrum of mutations or structural alterations predicted to activate the RAS-MAPK pathway^31,32^. A substantial array of therapeutic approaches to target the RAS-MAPK signaling is currently on the horizon for cancer therapy. A better understanding of how PRMTs modulate specific oncogenic pathways will provide insights into novel combinational therapies in high-risk and relapsed neuroblastomas, and possibly other cancers.

In summary, we have uncovered an essential survival pathway mediated by the PRMT1-ATF5 axis in neuroblastoma. PRMT1 promotes cell survival through epigenetic activation of the prosurvival factor ATF5, thus providing a new mechanistic link between overexpression of PRMT1 and compromised apoptotic pathway, a malignant phenotype characteristic of a large number of cancers, including neuroblastoma. We also assessed the therapeutic relevance of PRMT1 inhibition with diamidine compounds using multiple relevant in vitro and in vivo model systems. Collectively, our results reveal that PRMT1-depdenent survival pathway provides potential druggable targets for neuroblastoma.

## Materials and methods

### Reagents

Furamidine, pentamidine, hexamidine, and TC-E5003 were purchased from Tocris. Decamidine and SKLB639 were described previously^15,17^. Antibodies used in this study include PRMT1^3^, PRMT5 (Millipore), aDMA (Cell Signaling), sDMA (Millipore), MYCN^3^, TH (Millipore), PHOX2B (Abcam), PARP (Cell Signaling), ATF5 (Abcam), Flag (Thermo Fisher Scientific), and β-actin^3^.

### Cell culture

Human neuroblastoma cell lines KELLY, SK-N-BE(2)-C, SH-SY5Y, and SK-N-AS were purchased from Sigma and ATCC, respectively. SH-EP1 cells were generously provided by Dr. Karim Malik. All cell lines have been authenticated using STR profiling (Fragment Analysis Facility, Johns Hopkins University) and tested for mycoplasma using PCR Mycoplasma Test Kit I/C. Cell viability and cell proliferation were performed by using Alamar blue and trypan blue staining as previously described^3^. Caspase-3/7 activities were detected using a Caspase-Glo 3/7 Assay according to the manufacturer’s instruction. Alamar blue assay was performed in parallel to control for the differences in cell number.

### Plasmids, transfection, and viral infection

The inducible knockdown constructs were generated by cloning shPRMT1 sequences^3^ into pTRIPZ and pTER vectors^33^. ATF5 expression construct was made by cloning ATF5 cDNA^34^ to pOZ-FH-N vector. Transfection was carried out by using Nucleofector^3^. siRNA transfection was performed by using ON-TARGETplus siRNA pool (Dharmacon) and Lipofectamine® RNAiMAX (Thermo Fisher). The infectious viruses were produced by calcium phosphate transfection as previously described^8^. Cells were selected with 1 μg/mL puromycin 24 h post-transfection or post-infection. PRMT1 rescue construct was described previously^8^. Rescued cells were sorted by using anti-interleukin-2 receptor-conjugated Dynabeads as previously described^8^.

### Animals and primary sphere cell culture

All animal experiments were conducted under the approval of the University of Rochester Institutional Animal Care and Use Committee. *Th-MYCN* mice were obtained from NCI Mouse Repository and maintained in a 129X1/SvJ background. Genotyping was carried out as previously described^35^. Tumors were visualized by abdominal ultrasound using a Vevo 3100 Imaging System and MX550D transducer. 3D volume measurements were carried out using Amira 6.1 software. Ultrasound imaging analysis was made by individuals blinded to the genotype, appearance, and treatment history of the mice. Neuroblastoma spheres were isolated from tumors of *Th-MYCN* homozygous mice and sphere growth, sphere forming and syngeneic tumor transplantation assays were performed as described previously^7^.

Based on the power analysis, a group size of *n* = 10 will provide 90% power to detect a difference of 22% between two groups of mice with a 5% significance level (two-sided *t* test).

### Immunoblotting and immunohistochemistry

Immunoblotting and immunohistochemical studies were performed essentially as previously described^3,36^.

### RNA analysis

Total RNA was extracted using the RNeasy Plus mini kit and RT-qPCR was performed as previously described^3^. RNA-seq and transcriptome analyses were completed by the University of Rochester Genomics Research Center. Gene ontology (GO) enrichment analysis was carried out by using cluster Profiler (v3.8.1) and visualized by ggplot2 (v2.2.1) and GO plot (v1.0.2)^37,38^. A Benjamini–Hochberg adjust *P* value <0.05 and fold change cutoff as log2 ratio ≥1 were used for detection of differentially expressed genes.

### Chromatin immunoprecipitation (ChIP)

ChIP assays were performed as described previously^8^ using antibodies specific for PRMT1 or H4R3me2a (Active Motif). The primers used for ChIP-qPCR are ATF5 promoter (Forward: TGGGAAGGAAAGGCTCGGAT; Reverse: CGGCGACACTGTAGTGAGAA) and CITED2 promoter (Forward: CTCAGAAGAGCCCAGTGTAGCA; Reverse: GGATGAGGTATGTTGGAAAGCAGA).

### Statistical analysis

Statistics were determined using Prism 7 software. Data were presented as mean ± SD. *P*-values of <0.05 (*) and <0.01 (**) were considered statistically significant.

## Supplementary information

Supplementary figure legends

Supplementary figure 1

Supplementary figure 2

Supplementary figure 3

Supplementary figure 4
