# Supplementary material for: PRMT1 promotes neuroblastoma cell survival through ATF5
Source: Oncogenesis. 2020 May 15;9(5):50. doi: 10.1038/s41389-020-0237-9 (PMC7229216; doi:10.1038/s41389-020-0237-9)
Supplement: Supplementary file 1 — Supplementary figure legends [file 41389_2020_237_MOESM1_ESM.docx]

**Supplementary Figure 1. Murine neuroblastoma sphere cells derived from *Th-MYCN* tumors. A,** Representative phase-contrast image of *Th-MYCN* primary (1^st^) sphere cells. **B,** Representative phase-contrast images of serial sphere-forming assays of single cell-derived *Th-MYCN* secondary (2^nd^) sphere cells. Primary sphere cells were mechanistically dissociated into single cells by pipetting, and plated at clonal density of one cell/well into 96-well plates. Secondary spheres were assessed after 3 and 7 days in culture. **C,** Representative syngeneic tumor growth curves of two mice after subcutaneous injection of *Th-MYCN* primary sphere cells.

**Supplementary Figure 2. PRMT1 is essential for cell proliferation in *MYCN* non-amplified human neuroblastoma cells.** Western blot of *MYCN* non-amplified human neuroblastoma cell lines SK-N-AS (**A**) and SH-EP1 (**C**) stably expressing shPRMT1-1 or scramble. Cell proliferation assays of SK-N-AS (**B**) and SH-EP1 (**D**) stably expression shPRMT1-1 or scramble. Data are mean ± SD (n=3) relative to the scramble control. **E**, Cell cycle analysis of SK-N-BE(2)C cells expressing shPRMT1-D6 or scramble with or without Dox treatment. 5X10^3^ cells were plated in Corning 3603 black 96-well plates and incubated for 72 hr. Cell cycle analysis was performed by using the Click-iT™ Plus EdU Alexa Fluor™ 647 Imaging Kit (ThermoFisher Scientific, C10634) on Celligo.

**Supplementary Figure 3. Substrate scavenging in PRMT1 knockdown cells.** Western blot of Kelly (**A**), SK-N-BE(2)C (**B**), SK-N-AS (**C**), SH-EP1 (**D**) and SH-SY5Y (**E**) cells transfected with ON-TARGETplus human siPRMT1 pool (siPRMT1) or ON-TARGETplus Non-targeting pool (siScr) using specific antibodies against aDMA, sDMA, PRMT1, PRMT5 and β-actin.

**Supplementary Figure 4. The *in vivo* toxicity of diamidine compounds. A,** Schematic representation of progression of spontaneously developing neuroblastoma tumor in homozygous *Th-MYCN* mice. The numbers shown are postnatal days. The drug treatment started from P28 for 10 days. **B,** Percentage of weight change of mice treated with saline or furamidine (5, 10 or 15 mg/kg; n=5). **C**, The drug treatment started from P28 for 7 days. **D**, Percentage of weight change of mice treated with DMSO or decamidine (10 or 15 mg/kg; n=5). **E**, The drug treatment started from P28 for 10 days. **F**, Percentage of change in tumor volume in mice treated with DMSO or hexamidine (n=4). **G**, Percentage of change in SK-N-BE(2)C xenograft tumor volume in mice treated with DMSO or decamidine (n=5). **H**, The drug treatment started from P14 for 4 weeks. **I**, Percentage of weight change of mice treated with saline or furamidine (10 mg/kg; n=5).
