# Supplementary figures and images for: PRMT1 promotes neuroblastoma cell survival through ATF5
Source: Oncogenesis. 2020 May 15;9(5):50. doi: 10.1038/s41389-020-0237-9 (PMC7229216; doi:10.1038/s41389-020-0237-9)

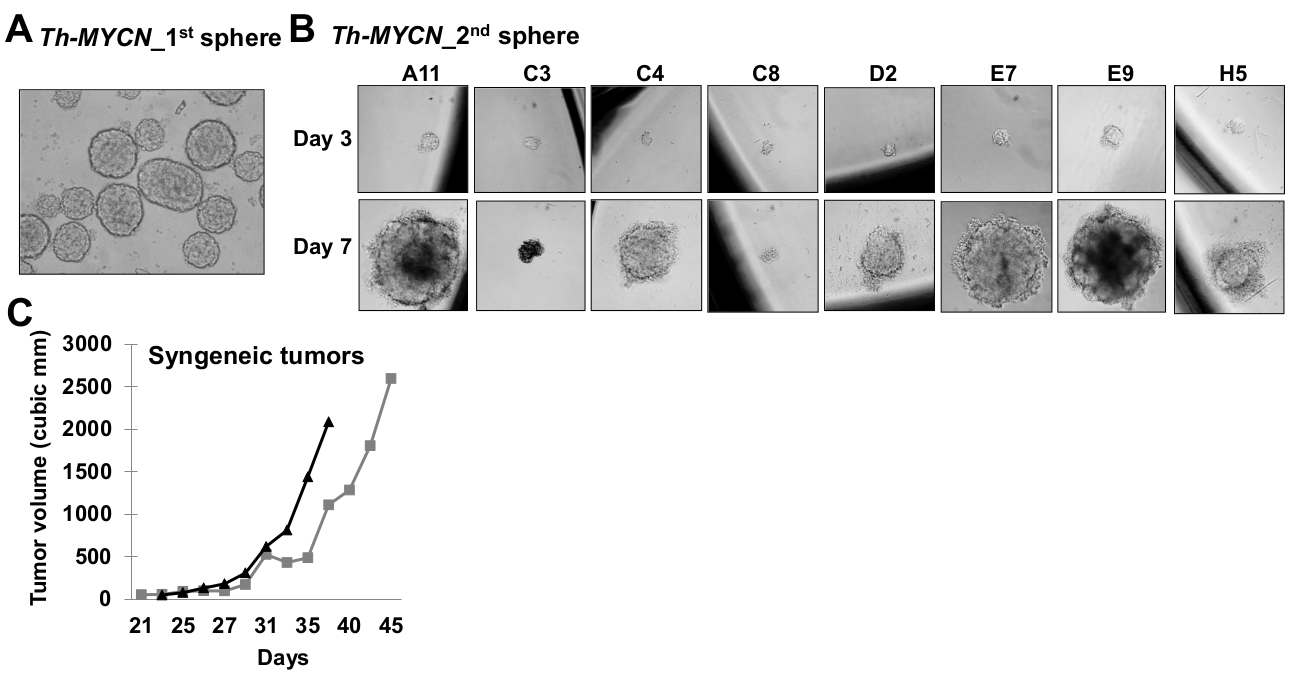

Supplement: Supplementary file 2 — Supplementary figure 1 [file 41389_2020_237_MOESM2_ESM.tif]

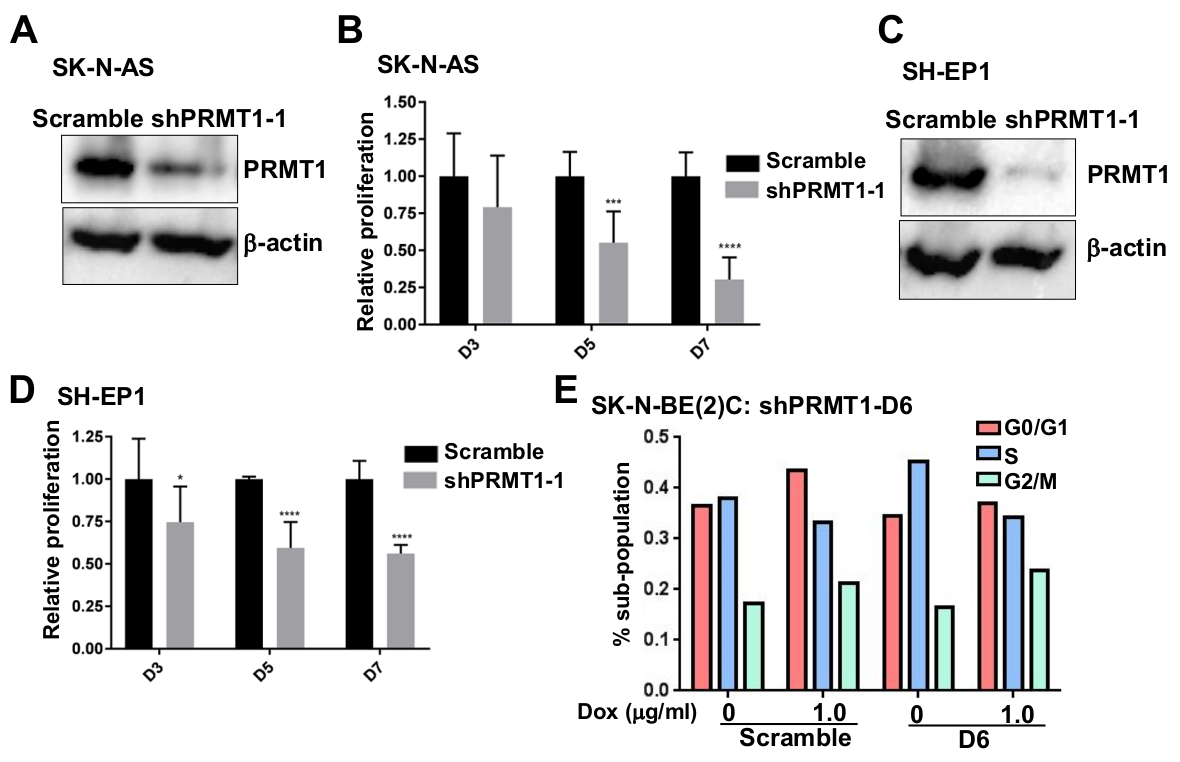

Supplement: Supplementary file 3 — Supplementary figure 2 [file 41389_2020_237_MOESM3_ESM.tif]

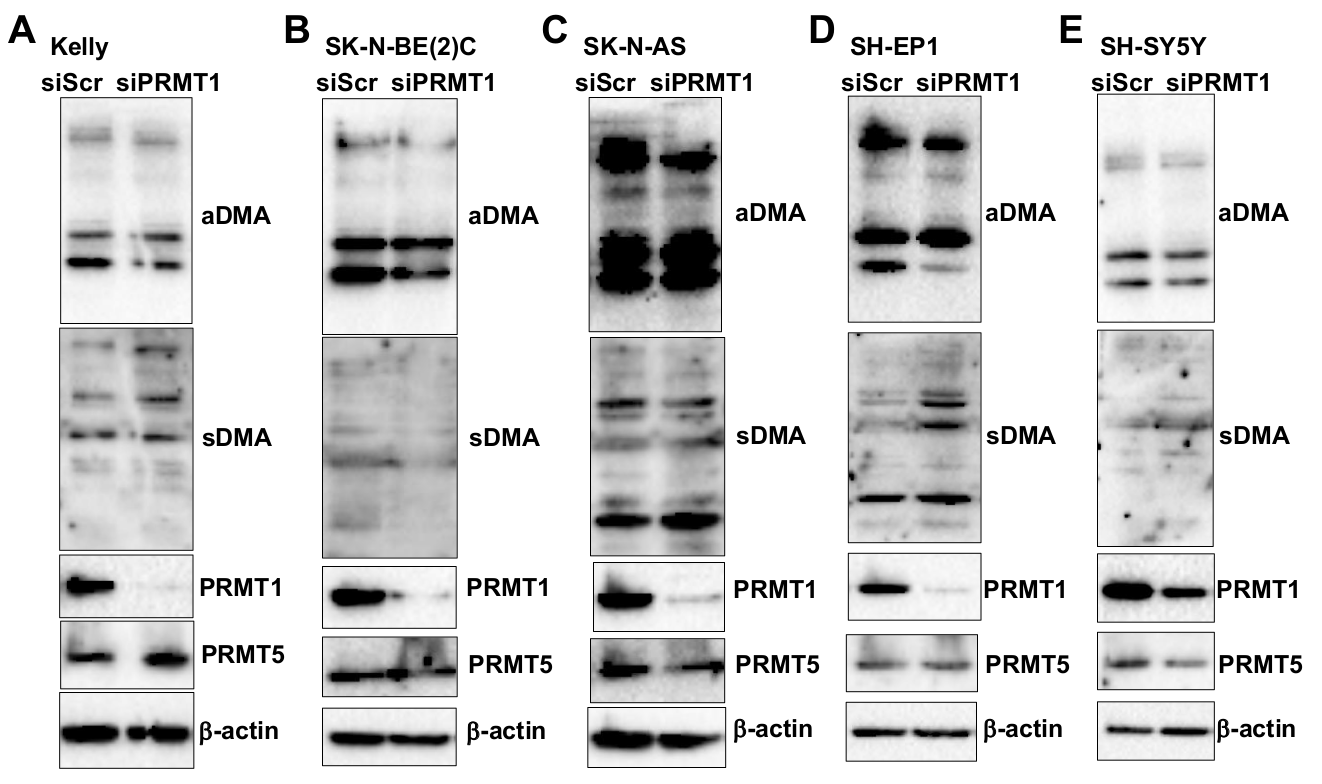

Supplement: Supplementary file 4 — Supplementary figure 3 [file 41389_2020_237_MOESM4_ESM.tif]

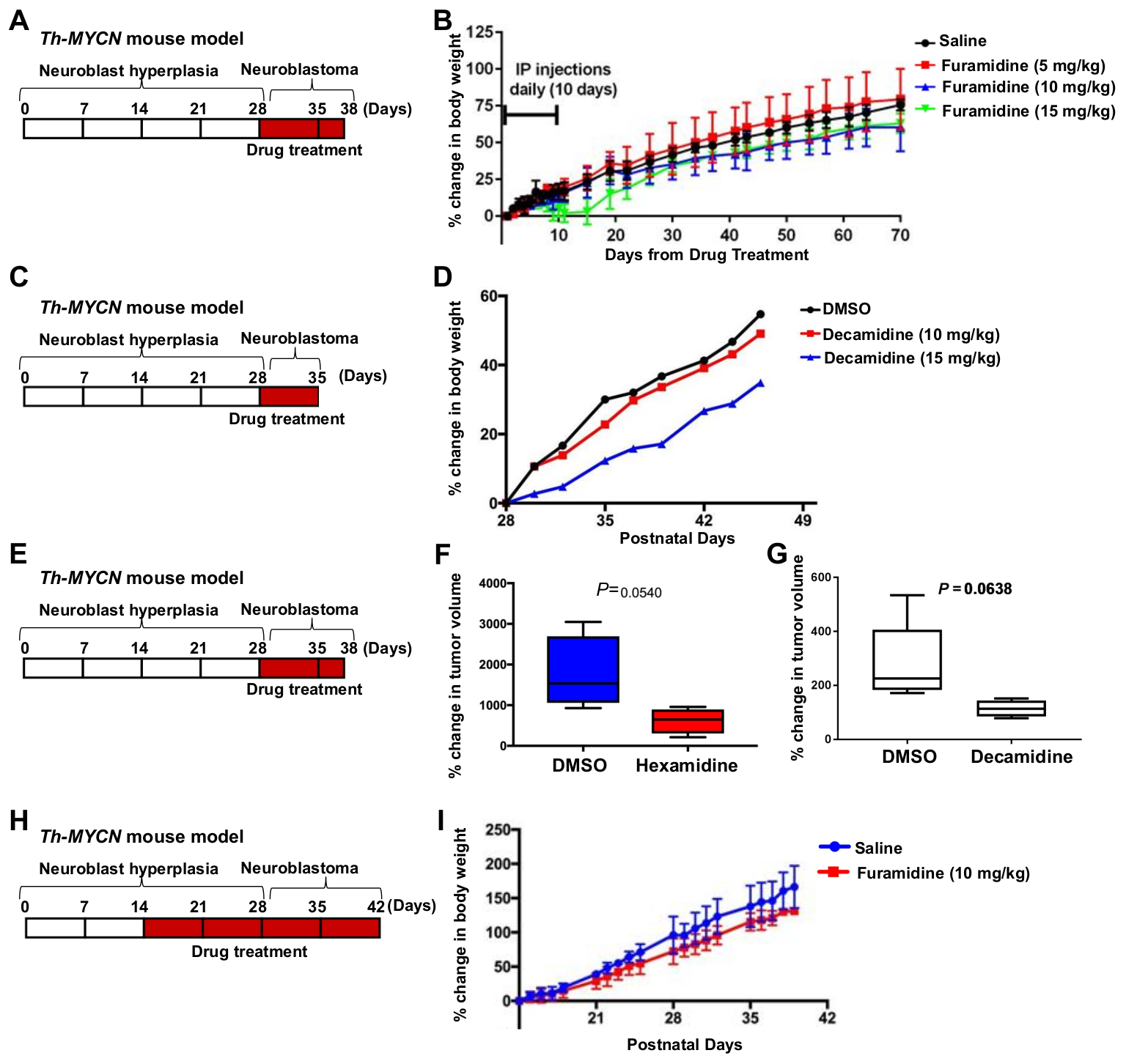

Supplement: Supplementary file 5 — Supplementary figure 4 [file 41389_2020_237_MOESM5_ESM.tif]
